# Potato tonoplast sugar transporter 1 controls tuber sugar accumulation during postharvest cold storage

**DOI:** 10.1093/hr/uhad035

**Published:** 2023-02-28

**Authors:** Tengfei Liu, Md Abu Kawochar, Shahnewaz Begum, Enshuang Wang, Tingting Zhou, Shenglin Jing, Tiantian Liu, Liu Yu, Bihua Nie, Botao Song

**Affiliations:** Key Laboratory of Horticultural Plant Biology, Ministry of Education, Key Laboratory of Potato Biology and Biotechnology, Ministry of Agriculture and Rural Affairs, College of Horticulture and Forestry Sciences, Huazhong Agricultural University, Wuhan 430070 China; Key Laboratory of Horticultural Plant Biology, Ministry of Education, Key Laboratory of Potato Biology and Biotechnology, Ministry of Agriculture and Rural Affairs, College of Horticulture and Forestry Sciences, Huazhong Agricultural University, Wuhan 430070 China; Bangladesh Agricultural Research Institute, Joydebpur, Gazipur 1701, Bangladesh; Key Laboratory of Horticultural Plant Biology, Ministry of Education, Key Laboratory of Potato Biology and Biotechnology, Ministry of Agriculture and Rural Affairs, College of Horticulture and Forestry Sciences, Huazhong Agricultural University, Wuhan 430070 China; Bangladesh Agricultural Research Institute, Joydebpur, Gazipur 1701, Bangladesh; Key Laboratory of Horticultural Plant Biology, Ministry of Education, Key Laboratory of Potato Biology and Biotechnology, Ministry of Agriculture and Rural Affairs, College of Horticulture and Forestry Sciences, Huazhong Agricultural University, Wuhan 430070 China; Key Laboratory of Horticultural Plant Biology, Ministry of Education, Key Laboratory of Potato Biology and Biotechnology, Ministry of Agriculture and Rural Affairs, College of Horticulture and Forestry Sciences, Huazhong Agricultural University, Wuhan 430070 China; Key Laboratory of Horticultural Plant Biology, Ministry of Education, Key Laboratory of Potato Biology and Biotechnology, Ministry of Agriculture and Rural Affairs, College of Horticulture and Forestry Sciences, Huazhong Agricultural University, Wuhan 430070 China; Key Laboratory of Horticultural Plant Biology, Ministry of Education, Key Laboratory of Potato Biology and Biotechnology, Ministry of Agriculture and Rural Affairs, College of Horticulture and Forestry Sciences, Huazhong Agricultural University, Wuhan 430070 China; Key Laboratory of Horticultural Plant Biology, Ministry of Education, Key Laboratory of Potato Biology and Biotechnology, Ministry of Agriculture and Rural Affairs, College of Horticulture and Forestry Sciences, Huazhong Agricultural University, Wuhan 430070 China; Key Laboratory of Horticultural Plant Biology, Ministry of Education, Key Laboratory of Potato Biology and Biotechnology, Ministry of Agriculture and Rural Affairs, College of Horticulture and Forestry Sciences, Huazhong Agricultural University, Wuhan 430070 China; Key Laboratory of Horticultural Plant Biology, Ministry of Education, Key Laboratory of Potato Biology and Biotechnology, Ministry of Agriculture and Rural Affairs, College of Horticulture and Forestry Sciences, Huazhong Agricultural University, Wuhan 430070 China

## Abstract

Cold-induced sweetening (CIS), the undesirable sugar accumulation in cold-stored potato (*Solanum tuberosum* L.) tubers, is a severe postharvest issue in the potato processing industry. Although the process of sucrose hydrolysis by vacuolar invertase during potato CIS is well understood, there is limited knowledge about the transportation of sucrose from the cytosol to the vacuole during postharvest cold storage. Here, we report that among the three potato tonoplast sugar transporters (TSTs), *StTST1* exhibits the highest expression in tubers during postharvest cold storage. Subcellular localization analysis demonstrates that StTST1 is a tonoplast-localized protein. S*tTST1* knockdown decreases reducing sugar accumulation in tubers during low-temperature storage. Compared to wild-type, potato chips produced from *StTST1*-silenced tubers displayed significantly lower acrylamide levels and lighter color after cold storage. Transcriptome analysis manifests that suppression of *StTST1* promotes starch synthesis and inhibits starch degradation in cold-stored tubers. We further establish that the increased sucrose content in the *StTST1*-silenced tubers might cause a decrease in the ABA content, thereby inhibiting the ABA-signaling pathway. We demonstrate that the down-regulation of β-amylase *StBAM1* in *StTST1*-silenced tubers might be directly controlled by ABA-responsive element-binding proteins (AREBs). Altogether, we have shown that *StTST1* plays a critical role in sugar accumulation and starch metabolism regulation during postharvest cold storage. Thus, our findings provide a new strategy to improve the frying quality of cold-stored tubers and reduce the acrylamide content in potato chips.

## Introduction

Sugar accumulation in the sugar-storing sink organs is a favorable trait that benefits productivity and quality in many crops, such as fruit, sugar beet taproots, and sugarcane stems. However, sugar, particularly reducing sugar (RS, mainly glucose and fructose), accumulation in potato (*S. tuberosum* L.) tubers is undesirable and impairs the tuber processing quality. Tubers are typically kept in cold conditions to minimize sprouting and disease. Unfortunately, low temperature stimulates starch breakdown leading to RS accumulation in tuber vacuoles, termed CIS. CIS is a persistent and critical issue in the potato processing industry. Upon processing at high temperatures, RS undergoes a nonenzymatic ‘Maillard reaction’ with free amino acids (mainly asparagine) to produce darker-colored products with potential carcinogenic acrylamide [1].

In the past decades, the mechanism of CIS in potato tubers has received much attention. Researchers have characterized several critical enzymes and regulatory proteins participating in CIS. For instance, inhibiting the α-glucan, GWD suppresses CIS [2]. Three amylase genes, *StAmy23*, *StBAM1*, and *StBAM9*, regulate CIS in distinct ways [3], while the amylase inhibitor SbAI contributes to CIS by restraining the amylase activity [4]. Three cytosolic glyceraldehyde-3-phosphate dehydrogenases play a redundancy function in CIS [5]. In the sucrose hydrolysis pathway, the vacuolar invertase is the chief factor in RS accumulation during CIS. Suppression of the invertase gene *StvacINV1*/*Pain-1* or overexpression of its inhibitor *StInvInh2B* can prevent CIS [6–9]. Moreover, the α and β subunits of Sucrose Nonfermenting1-Related Protein Kinase (SnRK1) form the protein complex with the invertase and its inhibitor to subtly regulate the acid invertase activity, thereby playing critical roles in CIS [10]. Even though the sucrose hydrolysis catalyzed by the vacuolar invertase during potato CIS is well characterized, little is known about how sucrose is transported from the cytosol to the vacuole during postharvest cold storage.

In sink organs, excessive sugars are typically synthesized as starch and stored in plastids or transported into vacuoles by tonoplast sugar transporters [11, 12]. TST 2.1 has been demonstrated to facilitate the accumulation of sucrose in sugar beet taproots [13]. Since then, an increasing number of TSTs have been shown to regulate sugar accumulation of sink organs, including CmTST2 from melon fruit [14], ClTST2 from watermelon [15], MdTST1 and MdTST2 from apple [16]. The above studies have shown that TSTs contribute to sugar accumulation of sink organs during development. However, it remains unclear whether TSTs are responsible for sugar accumulation in sink organs during postharvest storage.

Sugar transporters, in addition to transporting sugars, are thought to serve as sugar sensors. For example, in the yeast *Saccharomyces cerevisiae*, extracellular glucose concentration is sensed by the two distinct membrane-bound glucose receptors, Rgt2p and Snf3p [17]. The sensors are highly similar to hexose transporter but with an extended cytoplasmic C-terminus that facilitates signal transduction [18]. The Hxt1 protein from *Ustilago maydis* has a dual role, acting as a transporter and sensor [19]. There is no study to elucidate the signaling function of sugar transporters in plants. Due to the characteristic feature of possessing an elongated hydrophilic loop connecting the sixth and seventh transmembrane domains, TSTs are thought to be incorporated into the complex network of sugar sensing [20]. Therefore, TSTs-mediated transcriptional changes in plants deserve to be explored.

Our previous work found that the potato genome possesses three TST isoforms [21]. Suppression of the tuber-expressed TST3-type isoform *StTST3.2* causes a significant decrease in the RS content of tubers at harvest; however, it does not affect the accumulation of RS in cold-stored tubers [21]. Therefore, the other potato TSTs might facilitate the accumulation of RS in tubers during cold storage. To test this hypothesis, we aim to uncover which TSTs would contribute to RS accumulation in cold-stored tubers. By combining various techniques such as quantitative expression analysis, biochemical analysis, genetic transformation, plant physiology, and transcriptome analysis, we have characterized StTST1 as the critical player in potato tuber RS accumulation during postharvest cold storage.

## Results

### *StTST1* is the highest expressed TST-isoform in potato tubers during postharvest cold storage and encodes a tonoplast protein

**Figure 1:**
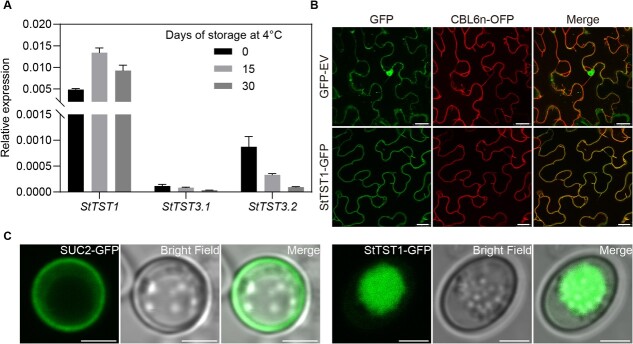
Expression analysis of potato *TST*s in tubers during postharvest cold storage and StTST1 subcellular localization determination. **A** The internal reference *ef1α* was used for *TST*s expression normalization, and the data depict the mean ± SD (*n* = 3). **B** and **C** Subcellular localization of StTST1 in *N. benthamiana* leaf cells (Bars, 20 μm) and yeast cells (Bars, 3 μm), respectively.

Our previous study showed that *StTST3.2* contributes to sugar accumulation in tubers at harvest; however, it is not involved in CIS [21]. We then analysed the expression pattern of *TST* genes in tubers from CIS-sensitive potato cultivar E3 during postharvest cold storage, as shown in Fig. 1A; it is not surprising that *StTST3.2* expressed low in tubers after cold storage. The expression levels of *StTST3.1* are extremely low in potato tubers at all storage stages, implying that *StTST3.1* might not function in tubers. The expression of *StTST1* in tubers is higher than that of *StTST3.1* and *StTST3.2*, and it was induced by cold storage, indicating that StTST1 might contribute to sugar accumulation in potato tubers during CIS.

We analysed the subcellular localization of StTST1 in *Nicotiana benthamiana* leaf cells through agroinfiltration. Unlike the free GFP showing no remarkable overlap with the tonoplast marker CBL6n-OFP [22], StTST1-GFP is located at the tonoplast, exhibiting considerable overlap with CBL6n-OFP (Fig. 1B). We further explored whether StTST1 could target the yeast membrane for transport activity analysis. As shown in Fig. 1C, the fluorescent signal of Arabidopsis SUC2 localizes to the cell membrane, while the fluorescent signal of StTST1 cannot; thus, it is unavailable for transport activity analysis of StTST1 in yeast.

### Suppression of *StTST1* has a limited effect on potato plant traits

**Figure 2:**
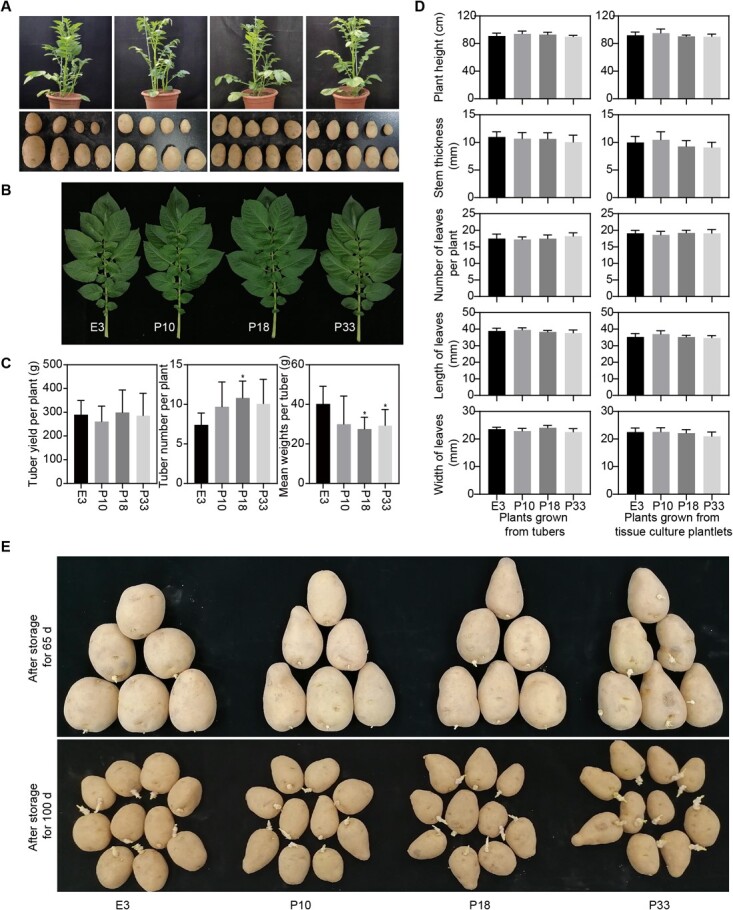
Plant traits of WT E3 and three *StTST1*-silenced lines. (**A**) Pictures show potato plants (60 d after transplantation of tissue culture plantlets to greenhouses) and tuber phenotypes. (**B**) Tuber yield, tubers number per plant, and mean weights per tuber. The data depict the mean ± SD (*n* = 6). ^*^ denotes significant differences at *P* < 0.05 between *StTST1*-silenced lines and WT E3 by Dunnett's multiple comparisons tests. (**C**) Pictures of leaves were captured 90 days after the transplantation of tissue culture plantlets to the greenhouse. (**D**) Plant height, stem thickness, number of leaves per plant, length of leaves, and width of leaves were measured from flowering plants grown from tubers and tissue culture plantlets, respectively. The data depict the mean ± SD (*n* = 10). (**E**) Tubers (over 10 tubers per line) were stored in darkness at 23°C for sprouting observation, and tubers photographs were captured after 65 and 100 d of storage.

To functionally characterize *StTST1* in potato, we developed over 20 *StTST1*-silenced transgenic lines in the background of E3. Three *StTST1*-silenced lines showing an approximately 80%–90% reduction in *StTST1* transcripts accumulation were selected for phenotyping (Fig. S1, see online supplementary material). We observed no significant differences in plants and tubers of the wild-type (WT) and transgenic plants grown in the greenhouse (Fig. 2A, B, and C). Plant traits were further analysed. *StTST1*-silenced lines showed no notable variation in terms of plant height, stem thickness, leaf size, and tuber yield per plant with WT (Fig. 2D). Compared with WT, *StTST1*-silenced lines showed slightly more tuber numbers but lighter mean tuber weights (Fig. 2B). In addition, there was no apparent difference in tuber sprouting between *StTST1*-silenced lines and WT (Fig. 2E).

### Suppression of *StTST1* dramatically inhibits RS accumulation in tubers during cold storage

**Figure 3:**
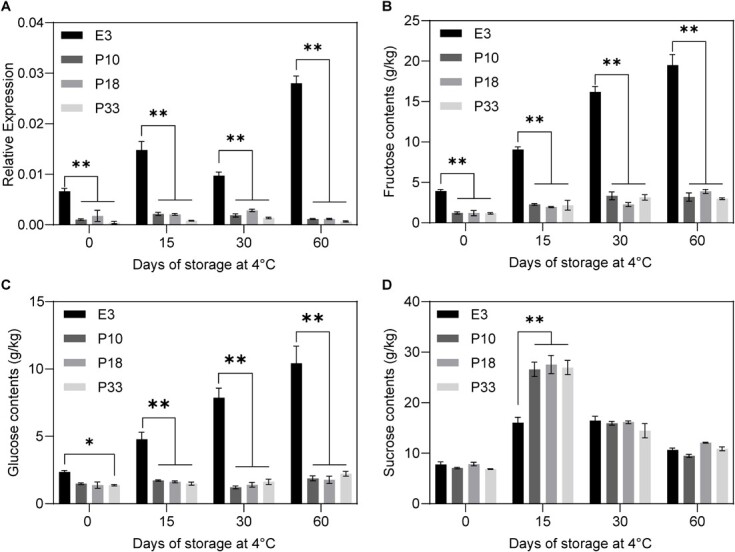
Relative expression levels of *StTST1* and sugar content in tubers from WT E3 and different *StTST1*-silenced lines. **A** The levels of *StTST1* transcripts were evaluated in E3 and transgenic tubers during storage at 4°C for different periods (0, 15, 30, and 60 days). The sugar content for fructose (**B**), glucose (**C**), and sucrose (**D**) of E3 and transgenic tubers during storage at 4°C for different periods (0, 15, 30, and 60 days). The data depict the mean ± SD (*n* = 3). ^*^ and ^**^ denote significance differences at *P* < 0.05 and *P* < 0.01 between *StTST1*-silenced lines and WT E3 by Dunnett's multiple comparisons test, respectively.

To assess the role of potato *StTST1* in CIS, the expression of *StTST1* and its association with sugar accumulation was studied by storing tubers at 4°C for varying periods (0, 15, 30, and 60 d). Compared with the WT control, *StTST1* expression exhibited sharp decreases in the three *StTST1*-silenced lines in all tested storage stages (Fig. 3A). Besides *StTST1* transcript abundance, the sugar content from the same tuber was quantified using HPLC-MS. RS, including fructose and glucose, increased rapidly after cold storage in tubers from E3 (Fig. 3B and C). In contrast to E3, *StTST1*-silenced tubers showed a relatively limited increase in RS content (Fig. 3B and C). Compared with WT plants, the RS content in *StTST1*-silenced tubers decreased significantly in all tested storage stages (Fig. 3B and C). Interestingly, *StTST1*-silenced tubers showed higher sucrose levels than WT tubers when stored at 4°C for 15 d (Fig. 3D), which might be because sucrose was less effectively transported to the vacuole. However, the sucrose content did not differ significantly when stored at 4°C for 30 d or 60 d between *StTST1*-silenced tubers and WT tubers (Fig. 3D), speculating that the high concentration of sucrose in the cytosol of *StTST1*-silenced tubers causes alterations in carbohydrate metabolism.

### Silencing of *StTST1* improves the quality of potato chips and decreases acrylamide levels

**Figure 4:**
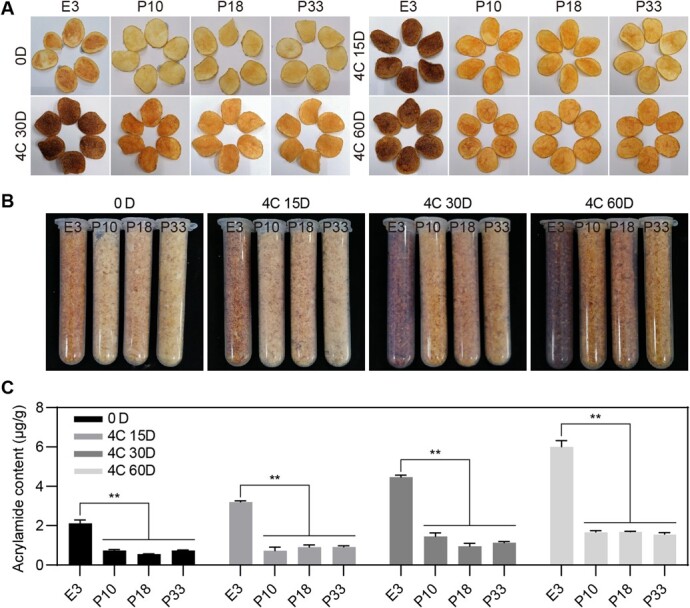
Processing quality analysis of cold-stored tubers. **A** Potato chips produced from tubers after storage at 4°C for 0 d, 15 d, 30 d, and 60 d, respectively. **B** Potato chips powders of potato chips displayed in **A**. **C** Acrylamide content of corresponding potato chips shown in **A**. The data depict mean ± SD (*n* = 3). ^**^ denotes a statistical significance at *P* < 0.01 between *StTST1*-silenced lines and WT E3 by Dunnett's multiple comparisons tests.

We next performed a chipping analysis of *StTST1*-silenced tubers at different storage stages. Consistent with the changes in RS content, with the extension of cold storage time, the color of potato chips processed from WT E3 gradually deepens; in contrast, those from *StTST1*-silenced lines only showed darker as storage time increases (Fig. 4A and B). Noticeably, potato chips produced from *StTST1*-silenced tubers showed visibly lighter color than those from WT tubers even after storage at 4°C for up to 60 d (Fig. 4A and B), which is further confirmed by color quantification using image processing (Fig. S2, see online supplementary material). As expected, the acrylamide content of *StTST1*-silenced potato chips exhibited a dramatic reduction compared with WT E3 at all tested storage stages (Fig. 4C). These findings indicated that *StTST1* knockdown could enhance the processing quality of cold-stored tubers and decrease acrylamide levels.

### Suppression of *StTST1* affects the expression of starch metabolism-related genes

**Figure 5:**
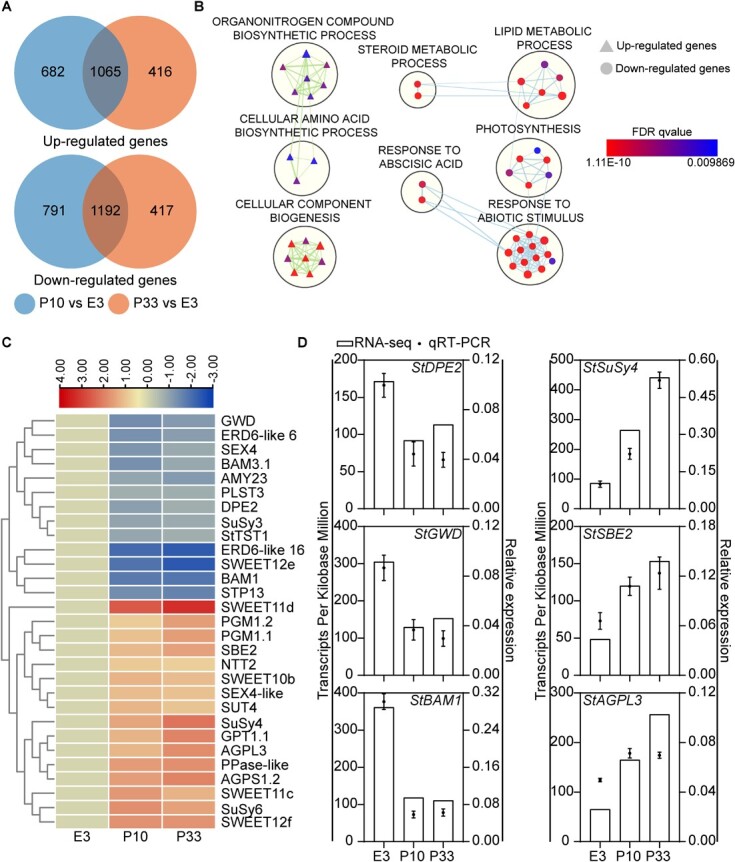
Transcriptome analysis of *StTST1*-silenced tuber after cold storage. **A** Venn diagram of up-regulated and down-regulated genes between tubers from *StTST1*-silenced lines and E3, respectively, when stored at 4°C for 15 d. **B** GO enrichment analysis for DEGs in tubers. The analysis only took into account terms related to biological processes. Each node represents a gene set that is significantly enriched (with a hypergeometric *P*-value less than 0.01). The edges indicate a degree of overlap as measured by a similarity coefficient. Highly similar gene sets are clustered by the MCL cluster algorithm. **C** Heatmap illustrates DEGs related to starch metabolism in *StTST1*-silenced (P10 and P33) tubers when stored at 4°C for 15 d based on RNA-seq. The level of change is represented as a log2 value, and the data in E3 is set as a baseline of 1 for each gene. **D** Quantitative RT-PCR validation of six DEGs related to starch metabolism identified by RNA-seq, including *disproportionating enzyme 2* (*StDPE2*), *glucan, and water dikinase* (*StGWD*), *β-amylase 1* (*StBAM1*), *sucrose synthase 4* (*StSuSy4*), *starch branching enzyme 2* (*StSBE2*), and *ADP-glucose pyrophosphorylase large subunit 3* (*StAGPL3*). The histograms depict RNA-seq results, as indicated by the Transcripts Per Kilobase Million (TPM) value. The black dots represent qRT-PCR results. Values are shown as the mean ± SD (*n* = 3).

Sucrose is not only a sugar metabolite but also an important signal molecule. We then performed RNA-seq to reveal the transcriptome changes after silencing *StTST1*. Regarding those *StTST1*-silenced tubers that accumulated the highest sucrose after cold storage for 15 d (Fig. 3D), total RNAs from tubers at this storage stage were employed for RNA-seq. Compared to the WT E3, 3730 and 3090 differentially expressed genes (DEGs) were identified in the P10 and P33 lines, respectively (Fig. 5A). Of these DEGs, 1065 up-regulated and 1192 down-regulated genes were common (Fig. 5A). The BiNGO enrichment generated a total of 47 significantly enriched BP gene sets, which formed eight distinguished clusters including ‘cellular component biogenesis’ and ‘response to abiotic stimulus’ for the most enriched up-regulated genes list and down-regulated genes list, respectively (Fig. 5B).

From the RNA-seq data, we found that compared to the WT E3, the expression levels of *StTST1* in P10 and P33 were significantly decreased, while the transcript abundance of *StTST3.1* and *StTST3.2* showed no significant change, indicating that the knockdown of *StTST1* by RNAi silencing is specific (Fig. S3, see online supplementary material). Similar to the results of qRT-PCR in Fig. 1A, Transcripts Per Kilobase Million (TPM) values of *StTST3.1* and StTST3.2 were much lower than those of *StTST1* in tubers after cold storage for 15 d (Fig. S3, see online supplementary material). After annotation, 29 DEGs were related to starch metabolism and sugar transporter genes (Fig. 5C; Data S1, see online supplementary material). Besides *StTST1*, five other sugar transporter genes were down-regulated, including *StSWEET12e*, *plastidic glucose transporter 3* (*StPLST3*), *sugar transport protein 13* (*StSPL13*), two *Glc exporter early response to dehydration like 6* (*StERDL6–6* and *StERDL6–16*). Five sugar transporter genes were up-regulated, including four *SWEET*s and *sucrose transport protein 4* (*StSUT4*) (Fig. 5C). For these starch metabolism-related genes, most up-regulated genes were synthesis-related, including *ADP-glucose pyrophosphorylase* genes (*StAGPL3* and *StAGPS1.2*), *sucrose synthase* genes (*StSuSy4* and *StSuSy6*), *inorganic pyrophosphorylase like* (*StPPase-like*), *nucleotide translocator 2* (*StNTT2*), *glucose 6-phosphate/phosphate translocator 1.1* (*StGPT1.1*), *phosphoglucomutase* genes (*StPGM1.1* and *StPGM1.2*), and *starch branching enzyme 2* (*StSBE2*) (Fig. 5C). In contrast, most starch degradation-related genes were down-regulated, including *amylase* genes (*StAmy23*, *StBAM1*, and *StBAM3.1*), *starch excess 4* (*StSEX4*), *disproportionating enzyme 2* (*StDPE2*), *glucan*, *and water dikinase* (*StGWD*) (Fig. 5C). Further qRT-PCR verification of the expression of six starch metabolism-related genes showed a consistent pattern with transcriptome data (Fig. 5D). Overall, these results implied that suppression of *StTST1* might alter carbohydrate metabolism by promoting starch synthesis and inhibiting starch degradation.

### Silencing of StTST1 might down-regulate the expression of *StBAM1* by inhibiting the ABA-signaling pathway

**Figure 6:**
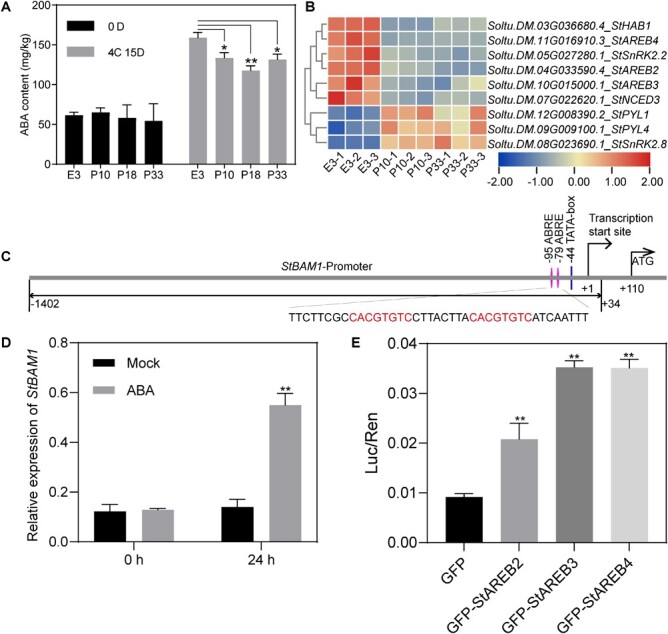
Down-regulation of the *StBAM1* expression in *StTST1*-silenced tubers was mediated by inhibiting the ABA-signaling pathway. **A** The ABA content of E3 and transgenic tubers when stored at 4°C for 0 and 15 d, respectively. **B** Heatmap illustrates DEGs related to the ABA pathway in *StTST1*-silenced (P10 and P33) tubers and WT E3 tubers when stored at 4°C for 15 d based on RNA-seq. **C** Schematic representation of the *StBAM1* promoter. The transcription start site of the promoter is assigned as +1. The −1402 to +34 regions of the *StBAM1* promoter were cloned and used to drive firefly luciferase. The positions of the ABA-responsive elements (ABREs) and TATA-box are indicated by pink and blue lines, respectively. The 40 bp sequence containing ABREs is annotated, and the ABREs are highlighted in red. **D** The expression of *StBAM1* was induced by ABA. **E** Transient dual-luciferase reporter assays show the StAREB2/3/4s’ ability to activate the promoter of *StBAM1* transcriptionally. The activities of firefly luciferase (LUC) and renillaluciferase (REN) were assessed sequentially, and the LUC/REN ratio was determined as the final transcriptional activation activity. The data depict as mean ± SD (*n* = 3). ^*^ and ^**^ denote significant differenced at *P* < 0.05 and P < 0.01 by Student's *t*-test, respectively.

Among these DEGs, *StBAM1* encodes active amylase, which plays a vital role in potato CIS [3]. Thus, we further investigated how *StBAM1* was down-regulated in the *StTST1-*silenced tubers. Given that a large number of down-regulated genes are enriched in ‘response to abiotic stimulus’ and ‘response to ABA’ (Fig. 5B), it raised the possibility that silencing of *StTST1* leads to inhibiting the ABA pathway. To test this hypothesis, we measured the ABA content in tubers. *StTST1*-silenced tubers accumulated significantly less ABA content than WT E3 after cold storage for 15 d (Fig. 6A). Coincidentally, several genes involved in the ABA pathway were down-regulated in *StTST1*-silenced tubers, including three AREB transcriptional factors (TFs), *StAREB2*, *StAREB3*, and *StAREB4* (Fig. 6B). We firstly cloned the promoter region of *StBAM1* and found that its promoter region contains two ABA-responsive elements (ABREs) (Fig. 6C), which have been demonstrated to be directly bound by StAREBs [23]. Therefore, it was speculated that these TFs might mediate the down-regulation of StBAM1 in the *StTST1-*silenced tubers. We examined and confirmed that *StBAM1* was induced by ABA treatment (Fig. 6D). The transient dual-luciferase reporter assay was further employed in *N. benthamiana* leaf cells to illustrate the three StAREBs’ ability to activate the promoter of *StBAM1* transcriptionally. We generated a dual-luciferase reporter vector containing a constitutive Renilla luciferase (REN) internal reference and firefly luciferase (LUC) driven by the *StBAM1* promoter. Compared to the vector control, the expression levels of *LUC* driven by the *StBAM1* promoter were significantly increased when co-transfected with the three GFP-StAREBs (Fig. 6E), suggesting that all the three StAREBs could transcriptionally activate the promoter of *StBAM1* in *N. benthamiana* leaf cells. The down-regulation of *StBAM1* in *StTST1*-silenced tubers might be primarily due to inhibition of the ABA signaling through these StAREBs.

## Discussion

As a complex and economically crucial postharvest trait, potato CIS has been extensively investigated in the past decades. Various strategies have been taken to reveal the mechanism for CIS, including the identification of starch-sugar metabolism-related genes and enzymes, comparative transcriptome analysis of CIS-sensitive and CIS-resistant potato tubers [24, 25], and intense mapping efforts that resulted in the discovery of multiple major quantitative trait loci (QTLs) [26, 27]. These studies expanded our knowledge of the mechanisms underlying CIS. The final step of tuber sugar accumulation during postharvest cold storage involves sugar uptake into vacuoles and hydrolysis by invertase to produce RS. However, these approaches have yet to identify the transporters responsible for sugar import into vacuoles during CIS. In the present study, we have demonstrated that the tonoplast sugar transporter *StTST1* contributes to potato CIS.

Of the three potato *TST* genes, *StTST1* has the most abundant transcripts in tubers; moreover, the expression of *StTST1* in tubers is induced by postharvest cold storage (Fig. 1). Therefore, it is not surprising that suppression of *StTST1* results in a dramatic reduction of RS during CIS (Fig. 3). Furthermore, from the recent transcriptomic data of CIS-sensitive and CIS-resistant potato tubers [24, 25], we found that *StTST1* transcript abundance in the cold-stored tubers from CIS-sensitive potato lines was higher than that from CIS-resistant lines after cold storage (Fig. S4, see online supplementary material), suggesting that low *StTST1* expression might be related to CIS resistance. Similar phenomena have been observed in other plant species. For example, the variation in sucrose accumulation of taproot between the two *Berberis vulgaris* varieties is also reflected in the transcript levels of *BvTST2.1* [13]. In melon, the expression of *CmTST2* is much higher in varieties with high sugar content compared to varieties with low sugar content [14]. In watermelon, the levels of *ClTST2* transcripts are positively related to fruit flesh sugar content; further molecular analysis indicates that the difference in *ClTST2* expression is due to the QTL causal SNP in the *ClTST2* promoter [15]. The chromosome location of *StTST1* is close to significant QTLs for tuber glucose and fry color in the overlapping region on chromosome 4 at 66.0 cM [26] . Future work will be interesting to uncover the relationship between these QTLs and *StTST1*.

During the CIS, sucrose is synthesized in the cytosol but degraded to hexoses in the vacuole by acid invertase [6]. RS accumulation is driven by dramatic increases in sucrose below 5°C [28]. Therefore, we speculate that sucrose is predominantly transported to the vacuole during CIS. In the present study, we have confirmed that StTST1 is a tonoplast-localized protein (Fig. 2). Suppression of *StTST1* causes a remarkable reduction in hexoses in cold-stored tubers and sucrose accumulation in tuber after cold storage for 15 d. These results indicate that StTST1 might function as a vacuolar sucrose transporter, consistent with sugar beet BvTST2.1 [13]. Moreover, in the *StTST1*-silenced tubers, the up-regulated expression of *CYCD2;1* (Soltu.DM.04G030670.1) and ribosome biogenesis-related genes implies an increased sucrose concentration in the cytosol of the transgenic tubers (Data S1, see online supplementary material), as sucrose enhances the expression of *CYCD2;1* and promotes ribosome synthesis [29, 30]. These findings indicate that silencing of *StTST1* might lead to sucrose retention in the cytosol, further confirming that StTST1 might serve as a tonoplast sucrose importer.

Sucrose is thought to be a signaling molecule to promote starch synthesis [31]. Sucrose may positively regulate potato tuber starch synthesis by activating the protein kinase StPKIN1 (Soltu.DM.03G029830.1) [32]. However, the expression of *StPKIN1* has no significant difference between tubers from *StTST1*-silenced lines and WT (Data S1, see online supplementary material), speculating that the activation of StPKIN1 by sucrose would be a post-transcriptional regulation. Several genes related to the starch biosynthetic pathway are induced by sucrose in different plant species [33, 34]. In the current study, several genes encoding starch synthesis-related enzymes were up-regulated in *StTST1*-silenced tubers with higher sucrose accumulation (Fig. 5). Three of them have already been functionally characterized in potato; for instance, StSuSy4 represents the main SuSy isoform in the tuber, and its activity correlates well with tuber starch accumulation [35]. *StAGPL3*, encoding a large subunit of the tetrameric enzyme that catalyzed the critical regulatory step in starch biosynthesis, is strongly inducible by sucrose [34]. Therefore, it is not surprising that the expression of *StAGPL3* is higher in *StTST1*-silenced tubers than in E3. *StNTT2*, encoding an ATP/ADP translocator, has been shown to contribute to tuber starch accumulation [36]. Additionally, the up-regulated Glc-6-phosphate/phosphate translocator *StGPT1.1* is associated with starch synthesis in heterotrophic tissues [37]. Accordingly, six starch degradation-related genes were down-regulated in *StTST1*-silenced tubers, including *StGWD*, *StAmy23*, and *StBAM1*, encoding glucan, water dikinase, α-amylase, and β-amylase, respectively, have been shown to play roles in potato CIS by regulating starch degradation [2, 3].

**Figure 7:**
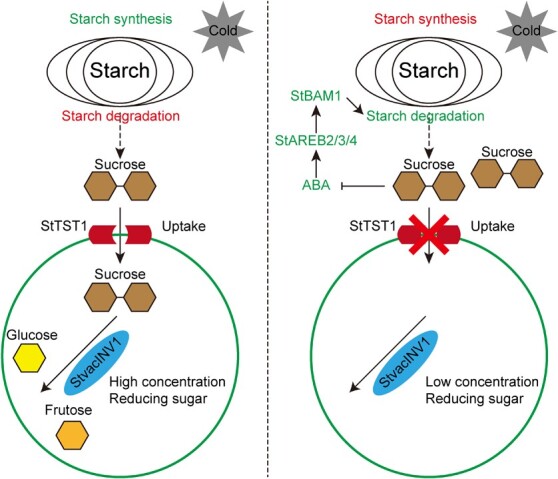
Proposed model of the role played by StTST1 during CIS.

A recent report has shown that a CIS-sensitive cultivar, Summer Delight, activates the expression of genes that participated in abiotic stress response and abscisic acid biosynthesis after cold storage [25]. In the current study, the enrichment analysis of the list of down-regulated genes shows that numerous genes are involved in ‘response to abiotic stimulus’ and ‘response to abscisic acid’ (Fig. 5). We confirm that *StTST1*-silenced tubers with higher sucrose content accumulate less ABA than WT tubers. The results suggest that the enhancement of CIS resistance by suppressing *StTST1* would be partially due to attenuated ABA-mediated stress response. Additionally, we have demonstrated that *StBAM1* is induced by ABA treatment. The promoter of *StBAM1* is directly activated by StAREB2/3/4, and genes encoding these proteins are all down-regulated in *StTST1*-silenced tubers with higher sucrose content (Fig. 6). Studies in *Arabidopsis thaliana* and apples have also shown that ABA treatment promotes starch degradation and sugar accumulation mediated by AREBs via directly controlling the transcription of genes related to starch-sugar metabolism. Therefore, it is rational to assume that cold induces the accumulation of ABA in tubers followed by activation of the downstream StAREBs, which can activate the expression of *StBAM1* by recognizing and binding ABRE in their promoter region, promoting starch degradation (Fig. 7). The association between ABA and CIS deserves further investigation. However, the possibility that *StBAM1* may be induced by sucrose independent of the ABA signaling pathway could not be ruled out.

During postharvest cold storage, cold stimulates starch degradation and RS accumulation in the vacuole by inducing starch degradation-related genes, tonoplast sugar transporter *StTST1*, and vacuolar invertase. When the *StTST1* was suppressed, sucrose could not load into the vacuole and be hydrolyzed by invertase, resulting in low RS content. Sucrose retention in cytosol might act as a signaling molecule to up-regulate starch synthesis-related genes and down-regulate starch degradation-related genes. Down-regulation of *StBAM1* in *StTST1*-silenced tubers might be due to the inhibition of ABA signaling caused by sucrose accumulation in the cytosol.

### Conclusions

*StTST1* is the most abundant *TST*-isoform in tubers during postharvest cold storage and encodes a tonoplast-localized protein. Suppression of *StTST1* has no noticeable impact on plant growth, tuber development, and tuber sprouting; however, it reduces RS accumulation, improves processing quality, and decreases acrylamide levels in cold-stored tubers. In addition, compromising tonoplast sugar transporter activity by silencing *StTST1* might result in sucrose retention in the cytosol, further redirecting the starch metabolism by promoting starch synthesis and inhibiting starch degradation. High sucrose content in cytosol might suppress ABA signaling by inhibiting ABA accumulation, thereby causing the down-regulation of β**-**amylase *StBAM1* in *StTST1*-silenced tubers. To summarize, our findings reveal that StTST1 is critical in sugar accumulation and starch metabolism regulation in cold-stored potato tubers.

## Materials and methods

### Quantitative real-time PCR and subcellular localization determination

The procedures of RNA extraction, cDNA synthesis, and qRT-PCR were performed as the previous description [21]. The specific sequences of primers utilized in the qRT-PCR analysis are detailed in Table S1 (see online supplementary material). To analyse StTST1 subcellular localization in planta, we generate the StTST1-GFP construct by cloning *StTST1* into pBI121-c-GFP. The StTST1-GFP fusion fragment was cloned into pDR196 for subcellular localization in yeast cells. Fluorescence observation and microscopy photography of StTST1-GFP in plant and yeast cells were conducted by the method described in the previous report [21].

### Plant transformation

The 354 bp DNA fragment from *StTST1* was amplified from the cDNA library of cultivar E-potato-3 (E3), which is sensitive to CIS, using specific primers (Table S1, see online supplementary material). Subsequently, the DNA fragment was purified and cloned into pHellsgate8 to produce the *StTST1*-RNAi vector. After Sanger sequencing confirmation, the construct was transferred into *Agrobacterium tumefaciens* strain GV3101. Then the cultivar E3 was infected by agrobacterium carrying *StTST1*-RNAi vector following the previous description [3].

### Plant growth conditions, tuber treatments, and potato chipping analysis

Potato plantlets (cultivar E3) were propagated on Murashige and Skoog's (MS) medium containing 3% (w/v) sucrose and incubated at 20 ± 1°C under a long day condition (16 h light/8 h dark) with a light intensity of 400 μmol m^−2^ s^−1^ in the growth chamber. For ABA treatment, 3-week-old *in vitro* cultivar E3 plantlets were treated with 50 μM ABA in 0.01%(v/v) Tween 20 or an identical solution without ABA (Mock treatment), starting 3 h after light exposure, samples were collected at 0 h or 24 h after treatment for further analyses. About 3-week-old plantlets were planted in pots with a diameter of 24 cm in the greenhouse (12 h light/12 h dark, light intensity range from 400 to 1000 μmol m^−2^ s^−1^, temperature 18–25°C). The method of counting the tuber number and measuring the weight of tubers was done according to the procedure previously described [21]. For postharvest cold storage experiments, tubers from the same line were combined and pre-stored at room temperature for two weeks in the darkness; after that, healthy tubers with similar size (around six for each time point) were picked randomly from the same line and stored at 4°C for up to 60 d. Tuber sampling and chipping analysis were performed according to a previous description [10]. For surveying tuber sprouting, over 10 healthy tubers of similar size for each line were stored at 23°C in the darkness to observe the sprouting state.

### Determination of sugar, acrylamide, and ABA

Potato tuber samples were powdered in liquid nitrogen and freeze-dried by the Labconco Freeze Dry System (Labconco, Kansas City, MO, USA). Approximately 30 mg of dried samples were utilized for extracting sugar and ABA. The process of extracting and measuring sugar followed the rapid and sensitive HPLC/MS method described by Georgelis *et al.* [38]. Potato chips produced from tubers stored at cold conditions for different stages were used for acrylamide extraction. The detailed procedure for acrylamide extraction and determination followed the previously described method [21]. Measurement of ABA content followed the method described by Pan *et al.* [39].

### mRNA-Seq and data analysis

About 2 μg RNA was used to construct the library. The method of constructing the library was executed as in the previous description [40]. The libraries were sequenced with an Illumina HiSeq 2500 platform producing 250 BP paired-end reads by Beijing Berry Genomics Biotechnology Co. Ltd. Clean reads were obtained following the method previously reported [40]. The improved DM1–3 v6.1 transcript reference was employed, and the transcripts were quantified using Salmon v.1.4.0 with default parameters [41, 42]. The R package DESeq2 [43] was used to normalize and analyse the DEGs. Significant DEGs were identified by setting the threshold of FDR <0.01 and the absolute value of the log2 (fold change) >0.75. GO enrichment analyses were conducted with the BiNGO plugin for Cytoscape software by setting the threshold of the Benjamini & Hochberg FDR <0.01 [44]. A Venn diagram and heat map were created by using TBtools software [45].

### Dual-luciferase reporter assay

A 1426 bp fragment (−1404 bp to +34 bp, containing two ABA-responsive elements) from the *StBAM1* promoter region was cloned into the dual-luciferase reporter vector pGreenII 0800. The *35*S:*GFP*-*StAREB2*, *35*S:*GFP*-*StAREB3*, and *35*S:*GFP*-*StAREB4* constructs, previously generated [23], were used as effectors. The agrobacterium containing reporter and effector plasmids co-infected *N. benthamiana* leaves. Each combination was tested using three biological replicates. After infection for 72 h, infected leaves were collected to measure LUC and REN activities using a dual-luciferase reporter assay system (E710, Promega, Madison, WI, USA). Transcriptional activation activity is indicated by the ratio of LUC to REN.

## Acknowledgments

This work was supported by the National Natural Science Foundation of China (31871683 and 32101781), the earmarked fund for the China Modern Agro-industry Technology Research System (CARS-09, Potato).

## Author contributions

B.N. and B.S. conceived the research. Te.L. and B.N. designed the study. Te.L., M.A.K., S.B., E.W., and T.Z. performed the experiments. Te.L., S.J., Ti.L., and L.Y. analysed the data. Te.L. drafted the manuscript. B.N. and B.S. revised the manuscript.

## Data availability

The data and materials used to support the findings of this study are available from the corresponding author upon request.

## Conflicts of interest statement

The authors declare no conflict of interest.

## Supplementary data

Supplementary data is available at *Horticulture Research* online.

## Supplementary Material

Web_Material_uhad035
