# Supplementary material for: Potato tonoplast sugar transporter 1 controls tuber sugar accumulation during postharvest cold storage
Source: Hortic Res. 2023 Feb 28;10(4):uhad035. doi: 10.1093/hr/uhad035 (PMC10548405; doi:10.1093/hr/uhad035)
Supplement: Web_Material_uhad035 [file web_material_uhad035.zip › supplementary data.docx]

# Potato tonoplast sugar transporter 1 controls tuber sugar accumulation during postharvest cold storage

Tengfei Liu^1^**†**, Md Abu Kawochar^1,2^**†**, Shahnewaz Begum^1,2^, Enshuang Wang^1^, Tingting Zhou^1^, Shenglin Jing^1^, Tiantian Liu^1^, Liu Yu^1^, Bihua Nie^1^*, Botao Song^1^*

1 Key Laboratory of Horticultural Plant Biology, Ministry of Education, Key Laboratory of Potato Biology and Biotechnology, Ministry of Agriculture and Rural Affairs, College of Horticulture and Forestry Sciences, Huazhong Agricultural University, Wuhan 430070 People's Republic of China

2 Bangladesh Agricultural Research Institute, Joydebpur, Gazipur 1701, Bangladesh

**†**These authors contributed equally to this work.

^*^Author for correspondence: Bihua Nie, [nbihua@mail.hzau.edu.cn](mailto:nbihua@mail.hzau.edu.cn); Tel and Fax: 86-027-87286946; Botao Song, [songbotao@mail.hzau.edu.cn](mailto:songbotao@mail.hzau.edu.cn); Tel and Fax: 86-027-87287381; Mailing address: No.1, Shizishan Street, Hongshan District, Wuhan, Hubei Province, 430070, P.R.China

E-mail address of other authors:

Tengfei Liu, [hzauzsmj@gmail.com](mailto:hzauzsmj@gmail.com);

Md Abu Kawochar, [kawocharpgrc@yahoo.com](mailto:kawocharpgrc@yahoo.com);

Shahnewaz Begum, [shahnewaz_ctg1952@yahoo.com](mailto:shahnewaz_ctg1952@yahoo.com);

Enshuang Wang, [wangenshuang@126.com](mailto:wangenshuang@126.com);

Tingting Zhou, [ttzh1989@126.com](mailto:ttzh1989@126.com);

Shenglin Jing: [jslmail20@sina.com](mailto:jslmail20@sina.com);

Tiantian Liu, [13296517556@163.com](mailto:13296517556@163.com);

Liu Yu, [214280485@qq.com](mailto:214280485@qq.com);.

**Table S1 Primers used in this research.**

| Primers | Sequence (5’-3’) | Related experiments |
| --- | --- | --- |
| qRTStTST1F | TATTGGTCGTCGCCCTATGC | qRT-PCR |
| qRTStTST1R | TAGGGGGACCAGAGTAACCG | qRT-PCR |
| qRTStTST3.1F | TCTGTTGCTTTGTCACGGGT | qRT-PCR |
| qRTStTST3.1R | TACACCAGCAAGTCCGATGG | qRT-PCR |
| qRTStTST3.2F | CACTGCTCTCACGTCAAGGT | qRT-PCR |
| qRTStTST3.2R | AATACCCATGCTGGCTTGCT | qRT-PCR |
| ef1αF | ATTGGAAACGGATATGCTCCA | qRT-PCR |
| ef1αR | TCCTTACCTGAACGCCTGTCA | qRT-PCR |
| RiStTST1F-Xba | CATTAAAGCAGGACTCTAGAGCAGGGTTGGGATAATGCTA | RNAi |
| RiStTST1R-Xba | ATAAGCTTGGATCCTCTAGAGGAAGAGTATTCAGTGACCC | RNAi |
| RiStTST1F-Xho | TTGGAGAGGACACGCTCGAGGCAGGGTTGGGATAATGCTA | RNAi |
| RiStTST1R-Xho | GGGGTACCGAATTCCTCGAGGGAAGAGTATTCAGTGACCC | RNAi |
| StTST1-GFPF | CGGGGGACTCTAGAGGATCCATGAATGGTGCTGTGTTAGT | Subcellular localization |
| StTST1-GFPR | CTGCCCCCTCCACCCTCGAGCTACTCATGCTTCGCGATAG | Subcellular localization |
| pDRStTS1-GFPF | TATACCCCAGCCTCGACTAGTATGAATGGTGCTGTGTTAGT | Yeast analysis |
| pDRStTST1-GFPR | HRYG3CQFARI72CUM2ESF8ZEXKQ5MAJE4VP9KHQBPGWZS | Yeast analysis |
| 0800-StBAM1pF | GGCGAATTGGGTACCTGAGGTGTACATGCCTAATT | Dual-luciferase reporter assay |
| 0800-StBAM1pR | TGGCGTCTTCCATGGAATGTGGAGAAGGTGGAGTT | Dual-luciferase reporter assay |
| qRTStAGPL3F | CGGGGAGAAGATCAGAGGGA | qRT-PCR |
| qRTStAGPL3R | CAGAGTAAGCAACCCCAGGT | qRT-PCR |
| qRTStBAM1F | TGAGATGCGTGACCATGAGC | qRT-PCR |
| qRTStBAM1R | CAAGTGGAACTTGCGCTTCC | qRT-PCR |
| qRTStDPE2F | AACATGCAATGACTGGCCTTT | qRT-PCR |
| qRTStDPE2R | CTGACTCGAGTTCTTCCTGACT | qRT-PCR |
| qRTStGWDF | GAATGGTCTTTTGCGTGATT | qRT-PCR |
| qRTStGWDR | TGTAGCCCATGCAGTTTGCA | qRT-PCR |
| qRTStSBE2F | CCCTGAGTGGATTGATTTCCCT | qRT-PCR |
| qRTStSBE2R | TTCTGCATCTCCCAGGTCAAA | qRT-PCR |
| qRTStSuSy4F | ATGAACCGAGTGAGGAATGG | qRT-PCR |
| qRTStSuSy4R | GCTGGACCACCGTGATTAGT | qRT-PCR |





**Figure S1** **Quantitative real-time PCR screening the *StTST1*-silenced transgenic lines.**

The values of the relative expression levels were normalized to *ef1α*, and the expression of *StTST1* in the wild-type E3 was taken as 1. The columns represent the mean values ± standard deviation (SD) (n = 3).


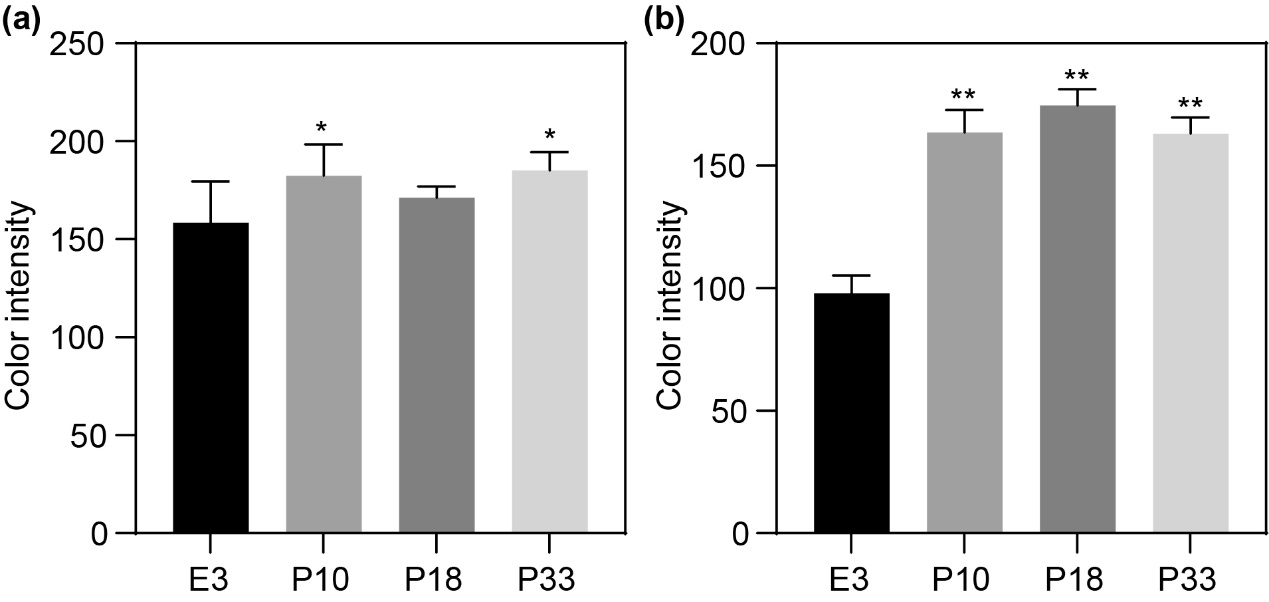


**Figure S2 Chip color quantification using image processing.**

The intensity in RGB color mode was measured for each image using the RGB measurement plugin in ImageJ, and color intensity was calculated as RGB values of (R+G+B)/3. (a) and (b) represented color intensity for chip color possessed from tubers after storage at 4°C for 0 d and 60 d, respectively. The columns represent the mean values ± SD (n=6). * and ** represent a statistical significance at P < 0.01 by Dunnett's multiple comparisons tests.


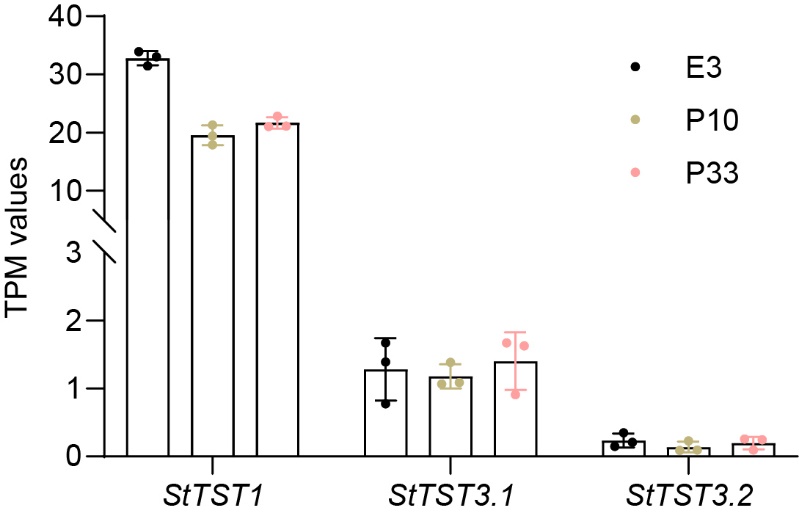


**Figure S3 The expression levels of *TST1s* in *StTST1*-silenced (P10 and P33) tubers and WT E3 tubers when stored at 4°C for 15 d based on RNA-seq.**

The columns represent the mean TPM values ± SD (n =3).


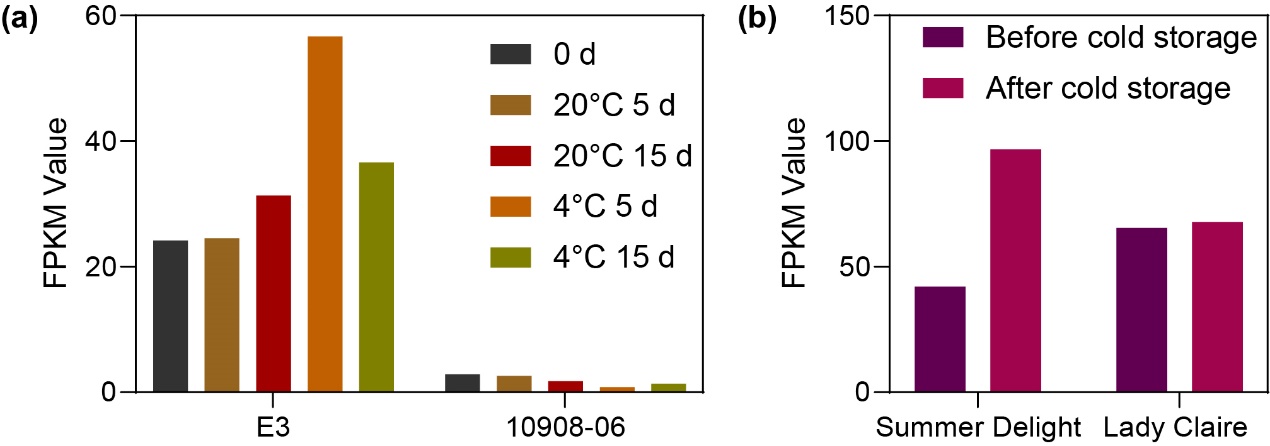


**Figure S4 The expression patterns of *StTST1* in tubers from different potato lines during storage by RNA-seq.**

(a) The expression of *StTST1* in tubers from CIS-sensitive potato E3 and CIS-resistant potato 10908-06 during storage. (b) The expression of *StTST1* in tubers from CIS-sensitive potato Summer Delight and CIS-resistant potato Lady Claire during storage.
